# Exercise stress CMR in patients with coronary heart disease - preliminary results

**DOI:** 10.1186/1532-429X-17-S1-P125

**Published:** 2015-02-03

**Authors:** Agnes Mayr, Markus Holotta, Regina Esterhammer, Klemens Mairer, Gert Klug, Hans-Josef Feistritzer, Bernhard Metzler, Michael Schocke

**Affiliations:** Radiology, Medical University Innsbruck, Innsbruck, Austria; Cardiology, Medical University Innsbruck, Innsbruck, Austria

## Background

Cardiac stress MRI has grown to a well established method to detect hypokinesia or perfusion deficits due to ischemia. Commonly, myocardial load is generated by the administration of adenosine or dobutamine, which is associated by a higher complication rate compared to exercise stress testing. Our purpose was to evaluate the use of an MR conditional pedal ergometer for cardiac stress MRI.

## Methods

We included 15 patients with coronary heart disease until now. All patients underwent exercise stress MRI at a whole-body 3 Tesla MR system and received real-time TRUFI CINE imaging and first-pass perfusion imaging before and after stress testing as well as late enhancement (LE) PSIR sequences after stress. All images were visually rated by three experienced radiologists.

## Results

As shown on LE images, two patients suffered from silent myocardial infarction and showed stress-induced hypokinesia and slight perfusion deficits nearby the infarction scar. In additional 5 patients, we detected segmental hypokinesia and endocardial perfusion deficits due to exercise. 3 patients exhibited endocardial perfusion deficits without hypokinesia. 5 patient did not show any abnormalities due to stress.

## Conclusions

Exercise stress of the myocardium is a helpful method to detect ischemic area in cardiac MRI.

## Funding

Nothing to declare.

